# Ectopic Adrenocorticotropic Hormone Production in a Stage IV Neuroendocrine Tumor: A Rare Presentation of Cushing's Syndrome

**DOI:** 10.7759/cureus.82689

**Published:** 2025-04-21

**Authors:** Faisal Aljabrain, Casey Strobelt, Benedict Cu, Annabelle Huntsman

**Affiliations:** 1 Department of Medicine, University of Utah Hospital, Salt Lake City, USA; 2 Division of Endocrinology, Department of Medicine, University of Utah Hospital, Salt Lake City, USA; 3 School of Medicine, University of Utah, Salt Lake City, USA

**Keywords:** adrenocorticotropic hormone (acth), autonomous cortisol secretion, cushing's syndrome, ectopic cushing's syndrome, neuroendocrine tumors (nets)

## Abstract

Neuroendocrine tumors (NETs) are heterogeneous neoplasms that arise from neuroendocrine cells, resulting in endocrine imbalances that impact quality of life and prognosis. Ectopic adrenocorticotropic hormone (ACTH) production by NETs is a rare cause of ACTH-dependent Cushing's syndrome. While the majority of these cases are associated with intrathoracic tumors, recent reports have indicated an increasing incidence of cases originating from diverse anatomical sites. Furthermore, despite comprehensive imaging efforts, a substantial proportion of cases remain challenging to localize.

In this case, we describe a 54-year-old man with a stage IV NET with metastatic liver and pancreatic lesions, who presented with Cushing's syndrome due to ectopic ACTH production. The patient exhibited symptoms of severe hypercortisolism, including weight gain, proximal muscle weakness, acute-onset heart failure, and hypertension. Imaging revealed bilateral adrenal hypertrophy. Laboratory tests revealed hypokalemia and hyperglycemia and confirmed elevated cortisol levels and a lack of suppression after dexamethasone administration, consistent with ectopic rather than pituitary ACTH production. The patient was treated with metyrapone because ketoconazole was contraindicated because of liver metastasis and recent upper gastrointestinal bleeding requiring proton pump inhibitor use. This case highlights the rare occurrence of ACTH-producing NETs and emphasizes the importance of considering this diagnosis in cases with similar presentations. Furthermore, medical management of this patient without surgical intervention, owing to multiple contraindications, offers an important perspective for treating complex cases.

## Introduction

Neuroendocrine tumors (NETs) are a heterogeneous group of neoplasms that can secrete various hormones; however, ectopic adrenocorticotropic hormone (ACTH) production is rare, occurring in only 5-10% of all Cushing's syndrome cases [1]. Liddle et al. described the first case in 1962 [2]. A recent case series that examined the clinical and diagnostic treatment of ectopic ACTH in a tertiary center included information on only 12 cases collected over a 17-year period [3]. The most common site for ectopic ACTH from malignancy is the intrathoracic region, primarily in small-cell lung carcinomas. Unfortunately, obtaining a single diagnostic image that can detect tumor-producing ACTH remains challenging. According to the literature, ectopic ACTH resulting in Cushing's syndrome can remain undetected [3,4].

In the present case, a patient with a stage IV NET presented with the classic features of Cushing's syndrome, leading to the diagnosis of ectopic ACTH production. The complexity of this case, owing to the patient's metastatic disease, the contraindications for certain therapies, and the requirement for atypical medical management, highlights the challenges of treating advanced NETs, especially metastatic lesions with hormonal overproduction. This report aimed to underscore the importance of early recognition and the effectiveness of metyrapone as a treatment for hypercortisolism in metastatic NET.

## Case presentation

**Figure 1:**
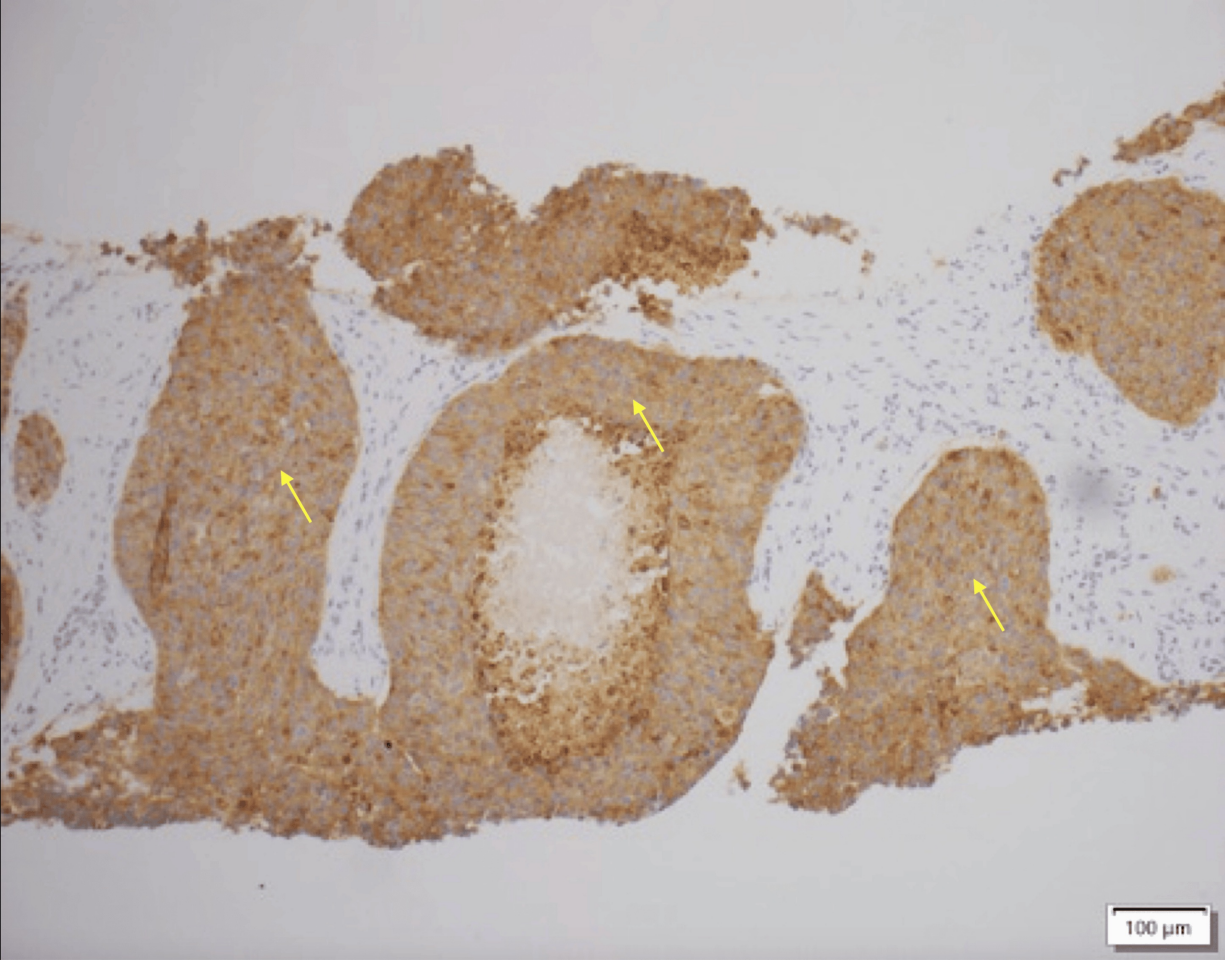
Liver tissue section showing positive synaptophysin immunohistochemical staining in neoplastic cells, consistent with a neuroendocrine neoplasm.

**Figure 2:**
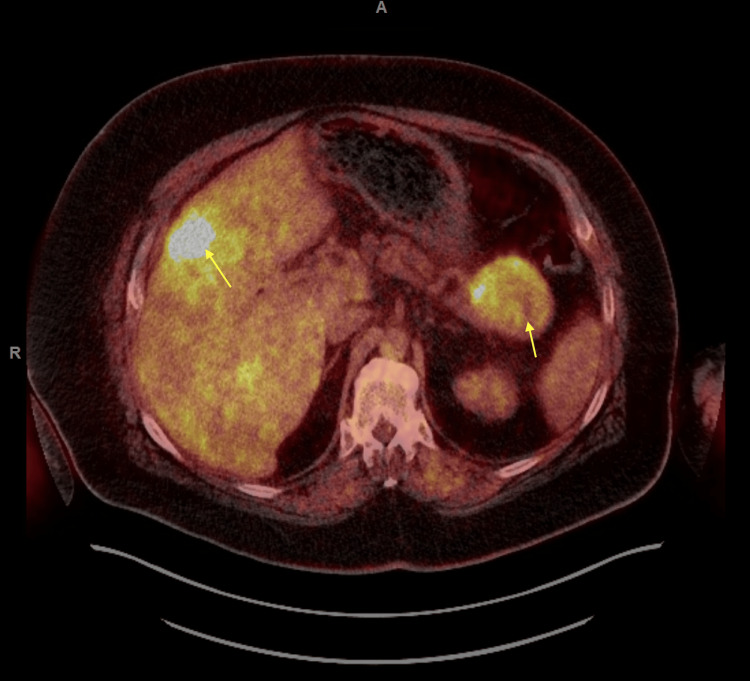
FDG PET/CT scan of the whole body showing hypermetabolic pancreatic tail mass which measures up to 6.5 cm and multifocal liver hypermetabolic metastases. FDG: fluorodeoxyglucose; PET: positron emission tomography; CT: computed tomography

A 54-year-old man with a known history of a World Health Organization (WHO) grade 3, stage IV NET with metastatic lesions in the liver and pancreas presented to the hospital with new-onset acute heart failure. His medical history consisted of papillary thyroid cancer diagnosed in January 2023, for which he underwent total thyroidectomy and left neck dissection. Three months later, the patient was found to have a new liver lesion that was biopsied and was consistent with a WHO grade 3 NET (Figure 1). He was started on capecitabine and temozolomide chemotherapy regimen, which was switched to folinic acid, fluorouracil, and oxaliplatin due to disease progression. He had undergone positron emission tomography (PET)/computed tomography (CT) as part of the follow-up for NET, and the findings were consistent with hypermetabolic pancreatic and liver lesions. However, no uptake was observed in the lungs and/or adrenal glands (Figure 2).

**Figure 3:**
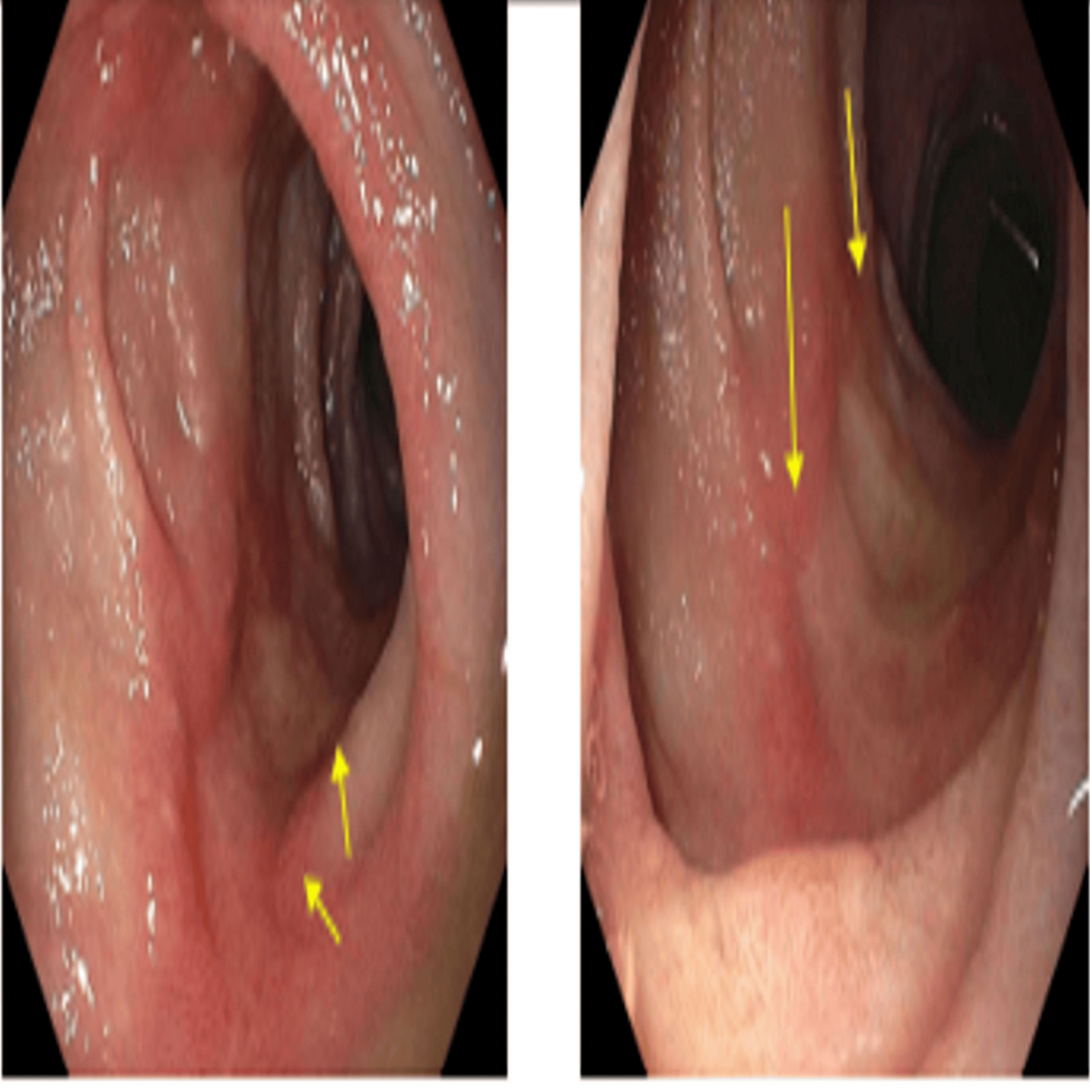
Upper endoscopy images showing four cratered, non-bleeding duodenal ulcers with a clean ulcer base (Forrest Class III).

**Figure 4:**
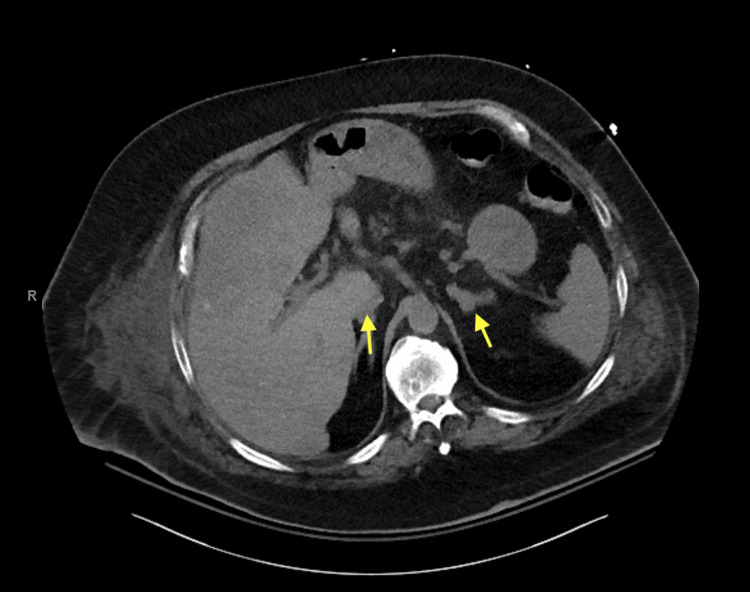
CT of the abdomen and pelvis demonstrating bilateral adrenal gland hypertrophy. CT: computed tomography

The patient was admitted first with gastrointestinal (GI) bleeding secondary to duodenal ulcers that were managed with a proton pump inhibitor (PPI), pantoprazole 40 mg, oral, BID (Figure 3). Ten days later, he presented with worsening dyspnea and shortness of breath, and clinical examination was consistent with volume overload and 4+ pitting edema in the lower extremities. Additionally, he was found to have a significantly low potassium level (2.6 mmol/L) and worsening serum blood glucose (341 mg/dL). The constellation of symptoms in the patient, including significant weight gain, obesity, easy bruising, proximal muscle weakness, acute-onset heart failure, hypertension, hypokalemia, and worsening hyperglycemia with new insulin requirements, raised concerns about hypercortisolism and prompted testing. The serum ACTH levels were markedly elevated (488 pg/mL; reference range: 10-60 pg/mL). CT of the abdomen and pelvis revealed bilateral adrenal gland hypertrophy (Figure 4).

Morning cortisol levels were significantly increased (42.2 µg/dL), and the 8-mg dexamethasone suppression test showed no suppression, with a post-dexamethasone cortisol level of 44.2 µg/dL. The 24-hour urinary-free cortisol level was elevated (2259 µg/24 hour; reference range: 3.5-45 µg/24 hour). At this time, the differential diagnoses included but were not limited to Cushing's disease or ectopic ACTH production secondary to metastatic NET. However, given that the patient had bilateral adrenal gland hypertrophy that was noted on imaging and his cortisol did not suppress with a high-dose dexamethasone suppression test, these findings support ectopic ACTH secretion secondary to metastatic NET over Cushing's disease from a pituitary source. 

**Figure 5:**
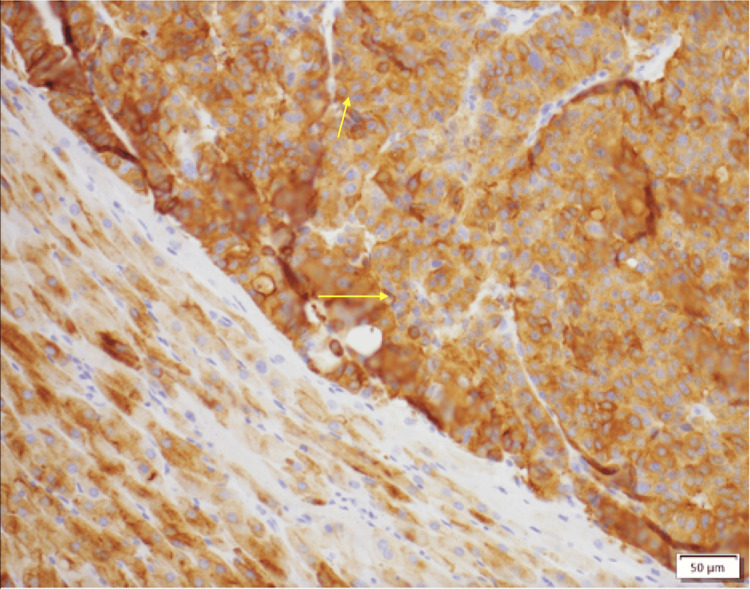
Adrenal tissue section showing positive synaptophysin immunohistochemical staining in neoplastic cells, consistent with a neuroendocrine neoplasm.

After confirming the diagnosis, the patient was started on metyrapone 500 mg, administered two times per day; his serum cortisol began to decrease (from 42 to 38 µg/dL) and continued to decline until it reached the lowest level (8.9 µg/dL) with metyrapone 500 mg, administered four times per day. Unfortunately, because of cost-related issues, the patient was switched to octreotide; however, subsequently, his serum cortisol level increased (from 8.9 to 49 µg/dL). Ketoconazole was not a viable option because of drug-drug interactions with PPI. Alternative suppressive medications were considered and included osilodrostat and mifepristone. However, given the patient's QTc prolongation and previous history of arrhythmia, it was felt that the use of these medications was too high risk for fatal arrhythmia. Given the limited medical options, the patient was evaluated for surgery, and, given the multiple comorbidities as well as metastatic disease without an apparent culprit lesion, he was not initially deemed to be a suitable surgical candidate. Therefore, metyrapone was reinitiated to control hypercortisolemia while the patient was admitted, and it effectively lowered his total serum cortisol levels. However, given that metyrapone was not a long-term option and medical management had failed (octreotide was ineffective in controlling serum cortisol levels, and ketoconazole could not be used due to drug-to-drug interactions with PPI), surgery was considered as an option. Despite the high risk associated with the procedures owing to the patient's condition, bilateral adrenalectomy was performed, considering the lack of medical options and the patient's goals of care. The patient was discharged home on oral hydrocortisone, 15 mg in the morning and 10 mg in the evening, in addition to fludrocortisone 0.1 mg daily. The patient's body surface area is 2.5 m². The pathology of his adrenal glands was consistent with that of a metastatic NET (Figure 5). The patient was seen in the endocrinology clinic after bilateral adrenalectomy for a follow-up almost one month after the procedure. He reported feeling tired and falling asleep quite often. He used to be able to walk; however, now, he could only make it a quarter of the way due to muscle weakness. Unfortunately, further follow-up and outcome could not be evaluated as the patient died three months after his bilateral adrenalectomy surgery, and the cause of death was unknown.

## Discussion

This case of a stage IV NET with ectopic ACTH production leading to Cushing's syndrome is notable because of its rarity and complexity. Although NETs are known for their diverse hormonal secretions, only a small subset of them are associated with ACTH production, making this case an important addition to the limited literature.

NETs causing ectopic Cushing's syndrome are most frequently found in the intrathoracic region (40-60%), including bronchial tumors, small-cell lung carcinoma, and thymic carcinomas. Additional sites where these tumors may occur include the pancreas and thyroid gland (particularly medullary thyroid carcinoma). Less common locations include the prostate, rectum, ovaries, and bladder [5].

Our patient's PET/CT findings were consistent with those of hypermetabolic lesions in the liver and pancreas. However, there was no uptake in the lungs, which is the most common site reported in the literature [5]. Additionally, there was no uptake in the adrenal glands, and the pathology was later found to be consistent with NETs. This posed a challenge to the diagnosis and identification of the culprit lesion. Reportedly, high-resolution cross-sectional CT imaging has a sensitivity of 50-67% in identifying the source of ectopic ACTH production, and when the findings are negative, a variety of nuclear medicine functional imaging techniques (Octreoscan, fluorine-18 fluorodeoxyglucose PET/CT, and gallium-68 somatostatin receptor-targeted PET/CT) can be used [6]. However, despite advances in imaging modalities, up to 20% of ectopic ACTH syndrome cases remain occult after initial imaging [4,7]. 

ACTH-producing pancreatic neuroendocrine (pNE) tumors are rare malignancies characterized by their aggressive nature [8]. Individuals diagnosed with this condition have less favorable outcomes compared with those with insulinoma, gastrinoma, or nonfunctional ACTH-producing pNE tumors [9]. The underlying reasons for the aggressiveness of the tumor and the resulting poor patient outcomes remain elusive. One study proposed that decreased methylation of the proopiomelanocortin promoter may enhance the ability of the tumors to secrete ACTH [10].

A similar presentation was reported by Al-Toubah et al. in a 2023 case series on ACTH-secreting pNE neoplasms. That study highlighted the rarity of ACTH production in these tumors and emphasized that such cases often present with severe hypercortisolemia and Cushing's syndrome. However, most patients in their series were treated with ketoconazole, which was not an option for our patient because of liver metastasis and recent upper GI bleeding requiring PPI treatment [11].

A systematic review published in February 2021 by Wu et al. investigated ACTH-producing pNE tumors. That study analyzed 210 publications, including data from 336 patients diagnosed with this condition. The results indicated a higher prevalence among female individuals (66.4%), at an average age of 44.7 years. The review reported the following frequencies of clinical symptoms: 69.3% experienced hypokalemia, 63.2% developed diabetes, 60.1% suffered from weakness, 56.4% had hypertension, 41.1% displayed moon face, and 37.4% presented with edema [12].

In the present case, the patient presented with decompensated heart failure, which is consistent with various case reports describing acute decompensated heart failure as the first presentation. Sugihara et al. reported three cases of Cushing's syndrome characterized by left ventricular failure as the predominant feature associated with gross left ventricular hypertrophy [13]. Similarly, Petramala et al. reported a case of a 28-year-old woman with Cushing's syndrome secondary to an adrenal adenoma who exhibited congestive heart failure as an initial symptom [14]. In this regard, some studies have examined the relationship between cardiac dysfunction and hypercortisolism and found that cardiac remodeling is independent of hypertension and is probably related to the direct action of cortisol on myocardial tissue via glucocorticoid receptors [15,16]. These cardiac impairments may be reversible with the appropriate treatment of the underlying hypercortisolism, such as the surgical resection of the adrenal adenoma or pituitary adenoma, and the medical management of heart failure [14].

Our patient received metyrapone and could not be treated using ketoconazole because of liver metastasis and drug-drug interactions with PPI, as previously mentioned. In 2022, Landry et al. studied the management of ACTH-secreting NETs [17]. Their study, including 76 patients, found that most patients had metastatic disease at the time of ectopic Cushing's syndrome diagnosis, similar to our case. Furthermore, they found that de novo hyperglycemia predicted worse survival outcomes. Therefore, controlling the hypercortisolic phase is crucial. Unfortunately, most patients present with metastatic disease, which makes surgical management, that is, removing the ACTH-producing tumor, not always an option. Additionally, they found that patients with medically resistant ectopic Cushing's syndrome, subsequently controlled with bilateral adrenalectomy, had significantly better disease-specific survival following ectopic Cushing's syndrome diagnosis than did patients who did not undergo bilateral adrenalectomy.

In our case, there were limited treatment options given the metastatic burden and limitations in using some of the medications to control hypercortisolism. In their article, Landry et al. stated "We have learned this over time as, unfortunately, most patients in our cohort who were diagnosed with resistant ectopic Cushing syndrome only used one type of suppression therapy by the end of the study" [17]. One medication, peptide receptor radionuclide therapy, was reported in multiple studies [5,18,19]. However, the Food and Drug Administration did not approve this therapy until 2018, and it has not been examined for ectopic Cushing's disease, especially in the metastatic NET setting.

As surgical resection remains the recommended first-line treatment for the majority of patients with Cushing's syndrome [20], medical therapy plays a critical role when surgery is not feasible; many studies reviewed the use of agents such as mifepristone [21], levoketoconazole [22], and pasireotide [23,24]. Additionally, a recent review study that focused on the clinical consideration for osilodrostat in the management of patients with ectopic ACTH found that quality of life improved during the use of long-term osilodrostat as a treatment for ectopic Cushing's syndrome raised from a pNE tumor [25].

## Conclusions

This case highlights the complexities involved in the diagnosis and management of ectopic ACTH-producing NETs. Due to the rarity of such presentations, clinicians must maintain a high index of suspicion for ectopic ACTH production in patients with unexplained hypercortisolism, particularly when signs of Cushing's syndrome are present. Additionally, the management of preoperative hypercortisolism may be challenging, as in our patient. The treatment approach in this case was unconventional, given the patient's ineligibility for surgery due to difficulties in localizing the exact lesion and the metastatic disease. Medical management with metyrapone was chosen. However, as it was cost-prohibitive, alternative therapy with octreotide was attempted, but it failed to achieve adequate control. Ketoconazole was not an option given the recent GI bleeding, and eventually, our patient underwent bilateral adrenalectomy. Therefore, future studies are required to develop predictive markers to determine which patients will benefit from bilateral adrenalectomy versus long-term pharmacotherapy. An extensive study on perioperative management in cases with ectopic ACTH would have proven to be useful in ensuring the survival of our patient.

## References

[1] Lacroix A, Feelders RA, Stratakis CA, Nieman LK (2015). Cushing's syndrome. Lancet.

[2] Liddle GW, Island DP, Ney RL, Nicholson WE, Shimizu N (1963). Nonpituitary neoplasms and Cushing's syndrome. Ectopic "adrenocorticotropin" produced by nonpituitary neoplasms as a cause of Cushing's syndrome. Arch Intern Med.

[3] González Fernández L, Maricel Rivas Montenegro A, Brox Torrecilla N (2023). Ectopic Cushing's syndrome: clinical, diagnostic, treatment and follow-up outcomes of 12 cases of lung ectopic ACTH. Endocrinol Diabetes Metab Case Rep.

[4] Varlamov E, Hinojosa-Amaya JM, Stack M, Fleseriu M (2019). Diagnostic utility of gallium-68-somatostatin receptor PET/CT in ectopic ACTH-secreting tumors: a systematic literature review and single-center clinical experience. Pituitary.

[5] Davi' MV, Cosaro E, Piacentini S (2017). Prognostic factors in ectopic Cushing's syndrome due to neuroendocrine tumors: a multicenter study. Eur J Endocrinol.

[6] Frete C, Corcuff JB, Kuhn E (2020). Non-invasive diagnostic strategy in ACTH-dependent Cushing's syndrome. J Clin Endocrinol Metab.

[7] Zisser L, Kulterer OC, Itariu B (2021). Diagnostic role of PET/CT tracers in the detection and localization of tumours responsible for ectopic Cushing's syndrome. Anticancer Res.

[8] Falconi M, Eriksson B, Kaltsas G (2016). ENETS consensus guidelines update for the management of patients with functional pancreatic neuroendocrine tumors and non-functional pancreatic neuroendocrine tumors. Neuroendocrinology.

[9] Maragliano R, Vanoli A, Albarello L (2015). ACTH-secreting pancreatic neoplasms associated with Cushing syndrome: clinicopathologic study of 11 cases and review of the literature. Am J Surg Pathol.

[10] Zhang C, Jin J, Xie J (2020). The clinical features and molecular mechanisms of ACTH-secreting pancreatic neuroendocrine tumors. J Clin Endocrinol Metab.

[11] Al-Toubah T, Pelle E, Hallanger-Johnson J, Haider M, Strosberg J (2023). ACTH-secreting pancreatic neuroendocrine neoplasms: a case-series. J Neuroendocrinol.

[12] Wu Y, Xiong G, Zhang H, Wang M, Zhu F, Qin R (2021). Adrenocorticotropic hormone-producing pancreatic neuroendocrine neoplasms: a systematic review. Endocr Pract.

[13] Sugihara N, Shimizu M, Shimizu K, Ino H, Miyamori I, Nakabayashi H, Takeda R (1992). Disproportionate hypertrophy of the interventricular septum and its regression in Cushing's syndrome. Report of three cases. Intern Med.

[14] Petramala L, Battisti P, Lauri G (2007). Cushing's syndrome patient who exhibited congestive heart failure. J Endocrinol Invest.

[15] Fallo F, Budano S, Sonino N, Muiesan ML, Agabiti-Rosei E, Boscaro M (1994). Left ventricular structural characteristics in Cushing's syndrome. J Hum Hypertens.

[16] Yiu KH, Marsan NA, Delgado V (2012). Increased myocardial fibrosis and left ventricular dysfunction in Cushing's syndrome. Eur J Endocrinol.

[17] Landry JP, Clemente-Gutierrez U, Pieterman CR (2022). Management of adrenocorticotropic hormone-secreting neuroendocrine tumors and the role of bilateral adrenalectomy in ectopic Cushing syndrome. Surgery.

[18] Cheung NW, Boyages SC (1992). Failure of somatostatin analogue to control Cushing's syndrome in two cases of ACTH-producing carcinoid tumours. Clin Endocrinol (Oxf).

[19] De Rosa G, Testa A, Liberale I, Pirronti T, Granone P, Picciocchi A (1993). Successful treatment of ectopic Cushing's syndrome with the long-acting somatostatin analog octreotide. Exp Clin Endocrinol.

[20] Gadelha M, Gatto F, Wildemberg LE, Fleseriu M (2023). Cushing's syndrome. Lancet.

[21] Fleseriu M, Molitch ME, Gross C, Schteingart DE, Vaughan TB 3rd, Biller BM (2013). A new therapeutic approach in the medical treatment of Cushing's syndrome: glucocorticoid receptor blockade with mifepristone. Endocr Pract.

[22] Fleseriu M, Auchus RJ, Pivonello R, Salvatori R, Zacharieva S, Biller BM (2021). Levoketoconazole: a novel treatment for endogenous Cushing's syndrome. Expert Rev Endocrinol Metab.

[23] Colao A, De Block C, Gaztambide MS, Kumar S, Seufert J, Casanueva FF (2014). Managing hyperglycemia in patients with Cushing's disease treated with pasireotide: medical expert recommendations. Pituitary.

[24] Trementino L, Cardinaletti M, Concettoni C, Marcelli G, Boscaro M, Arnaldi G (2015). Up-to 5-year efficacy of pasireotide in a patient with Cushing's disease and pre-existing diabetes: literature review and clinical practice considerations. Pituitary.

[25] Fleseriu M, Auchus RJ, Bancos I, Biller BM (2025). Osilodrostat treatment for adrenal and ectopic Cushing syndrome: integration of clinical studies with case presentations. J Endocr Soc.

